# The Gastric Obstruction Due to Orbeez Beads Ingestion: A Case Report With Esophagogastroduodenoscopy Findings

**DOI:** 10.7759/cureus.51857

**Published:** 2024-01-08

**Authors:** Marjan Haider, Aamir Saeed, Michael Zijlstra, Kevin Wenzke, Emily Tommolino

**Affiliations:** 1 Internal Medicine, St. Joseph Mercy Ann Arbor Hospital, Ann Arbor, USA; 2 Internal Medicine, Merit Health Wesley Hospital, Hattiesburg, USA; 3 Gastroenterology and Hepatology, Trinity Health Ann Arbor Hospital, Ann Arbor, USA

**Keywords:** esophagogastroduodenoscopy, gastrointestinal tract, water-absorbing beads, orbeez beads, foreign bodies

## Abstract

Ingestion of non-food entities poses a critical risk, particularly in children and young adults. Mostly foreign bodies can safely pass through the gastrointestinal tract if they traverse the pylorus; however, ingestion of Orbeez beads can present as a unique challenge. Orbeez beads have the potential to absorb water and can expand in the stomach and small intestine, and can result in complications including constipation, intestinal obstruction, perforation, and peritonitis. Timely diagnosis and management are crucial to improve patient outcomes. We present a case of a 19-year-old male who ingested Orbeez beads and presented with nausea, vomiting, and abdominal pain. A non-contrast CT scan of the abdomen confirmed the foreign bodies. Fifty to seventy beads were successfully removed via esophagogastroduodenoscopy (EGD) without any complications, and the patient is currently doing well.

## Introduction

Foreign body ingestion and food bolus impaction are frequent occurrences, with a yearly report of over 100,000 cases in the United States, predominantly involving the pediatric population [1]. The most commonly ingested foreign objects are coins, toys, magnets, batteries, and safety pins. While most of these objects pass through the gastrointestinal tract (GIT) spontaneously, around 20% may require endoscopic intervention, and less than 1% may need surgical intervention [2]. Orbeez beads are made of a super-absorbent polymer, specifically sodium polyacrylate. Upon contact with water, these beads can enlarge significantly, posing a potential choking hazard or causing blockages in the digestive tract if ingested [3].

Accidentally ingested beads are typically found in the upper GIT at the time of presentation, with rare cases leading to obstruction in the jejunum, ileum, and colon. The timing of endoscopic intervention in water-absorbing beads is determined by the risks of aspiration, obstruction, and perforation [4]. Patients with a history of GIT surgery or congenital gut malformations are at a higher risk of obstruction and perforation [2,4]. The clinical presentation varies based on the level of obstruction. This can include drooling and dysphagia at the level of the esophagus, as well as nausea, vomiting, abdominal distension, and abdominal pain with small bowel obstruction. Plain radiography is recommended as the initial diagnostic modality in patients with suspected foreign body ingestion, and computed tomography (CT) scans are usually applied when there is perforation or surgical intervention is needed [3,4]. Endoscopic intervention is necessary due to the water-absorbing ability of Orbeez beads, which can cause an increase in size. Surgical removal is warranted in cases of obstruction. We present a young patient who had vomiting and generalized abdominal pain after ingesting numerous Orbeez beads.

## Case presentation

**Figure 1:**
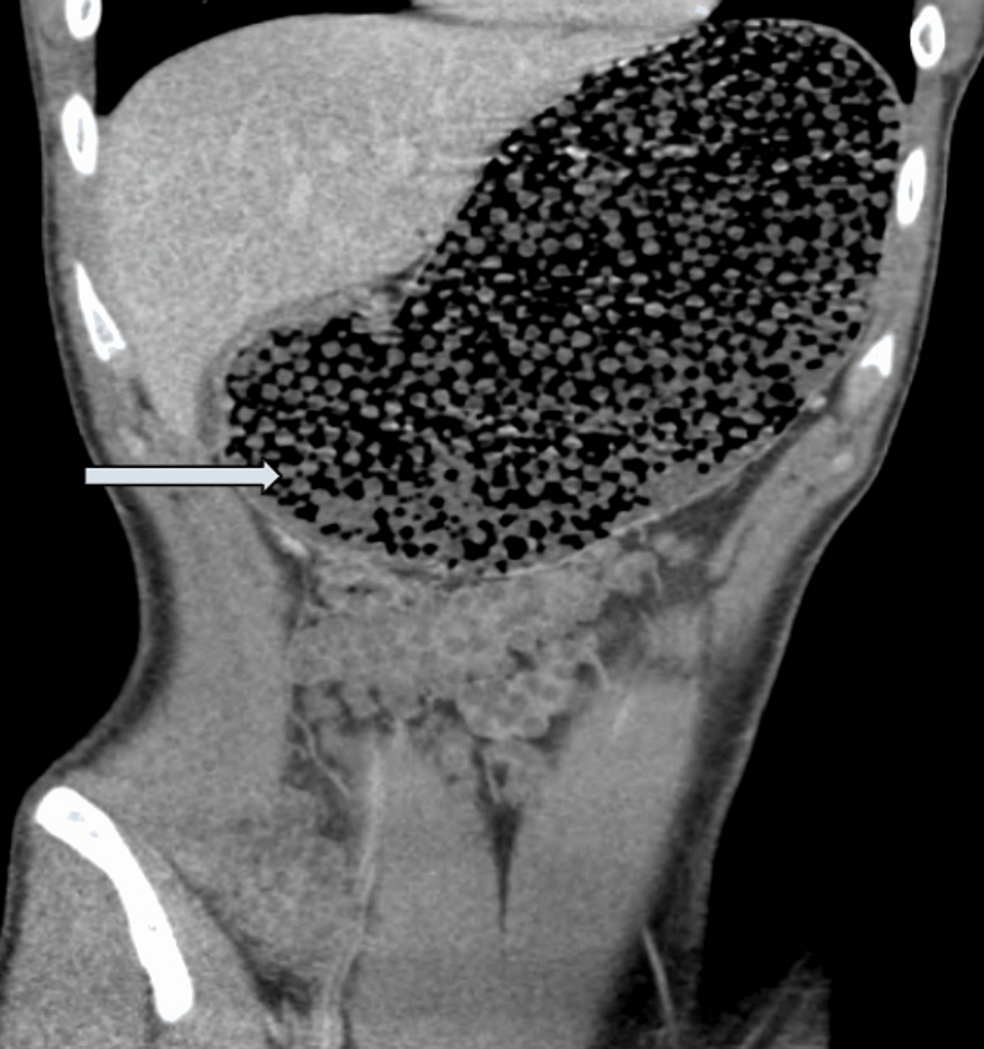
Unenhanced CT scan of the abdomen and pelvis showing innumerable small, rounded densities diffusely throughout the GI tract, most pronounced in the stomach (arrow). GI: gastrointestinal

A 19-year-old male with no past medical history presented to the emergency department with nausea, vomiting, and abdominal distention about 30 minutes after the incidental ingestion of a large amount of Orbeez beads. His presenting vitals were blood pressure of 127/77 mmHg, heart rate of 104 beats per minute, oxygen saturation (SpO2) of 100%, respiratory rate of 18 breaths per minute, and temperature of 98.2 F. Initial blood work including complete blood count, complete metabolic profile, cardiac enzymes, and serum lipase level were unremarkable. Furthermore, a CT scan of the abdomen and pelvis without contrast showed innumerable small, rounded densities diffusely throughout the GIT, most pronounced in the stomach (Figure 1).

**Figure 2:**
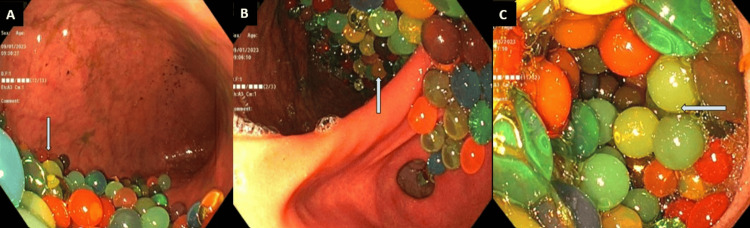
Esophagogastroduodenoscopy (EGD) which showed LA grade B esophagitis along with innumerable small colored balls (Orbeez) found in the gastric body (A & B) and fundus (C).

The patient underwent an emergent esophagogastroduodenoscopy (EGD) which showed LA grade B esophagitis along with innumerable small colored balls (Orbeez) found in the gastric body and fundus (Figures 2A, 2B, 2C).

The endoscope was removed, and an overtube with the cap was fitted. The scope and overtube were then reinserted and advanced to the esophagus to aid in foreign body removal. Removal of around 50 to 75 beads was successful using a Roth Net® (STERIS, Mentor, USA) but numerous (at least 200 to 300) were remaining. Numerous Orbeez were also found in the duodenal bulb and the second part of the duodenum. Orbeez beads were visualized to be small in size on EGD and were thought to most likely pass on their own with supportive management. Removal of 200-300 more beads via Roth Net would have taken hours, so complete removal was not attempted. The patient tolerated the procedure well and was admitted to monitor for the passing of foreign bodies. During hospitalization, on obtaining a detailed history, the patient reported, waking up at night and unintentionally ingesting beads by drinking from a water bottle. The patient was concurrently being evaluated by a psychiatrist, who suggested unintentional foreign body ingestion, and currently without any suicidal ideation or intent.

The patient was kept on polyethylene glycol 3350 to assist with the passing of remaining foreign bodies. The patient's symptoms were improving, he was passing the beads in his stools, and the X-ray of the abdomen showed the progression of innumerable round foreign bodies, measuring up to 0.7 cm in size, from the stomach and small intestine to the colon. After the endoscopy, he experienced multiple bowel movements, with Orbeez beads passing. The patient was discharged with a prescription for polyethylene glycol 3350 for seven days. He was instructed to return to the emergency department in case he experienced a recurrence of symptoms, such as abdominal pain, nausea, or vomiting.

## Discussion

The ingestion of foreign bodies, such as non-food objects, is more common in children than in adults. In adults, food bolus impaction in the esophagus is a prevalent issue, with an annual incidence of 13 cases per 100,000 people [5]. Water-absorbing beads present a unique problem due to their ability to grow in size up to 200 times, with the normal size of the bead typically ranging from 2 to 4 mm. Small beads may gradually increase in size during their passage through the digestive tract, leading to complete obstruction [6]. Orbeez bead ingestion usually obstructs areas of physiological narrowing, such as the middle one-third of the esophagus, lower esophageal sphincter, pylorus, and ileocecal valve. A study by Arana et al. involving 325 pediatric patients revealed that most foreign bodies were found in the stomach, followed by the esophagus, small intestine, and oropharynx [7]. The study by Forrester, comprising a case series of 110 patients, revealed that the predominant adverse effects linked to superabsorbent polymer toys encompassed vomiting, constipation, abdominal pain, and fever. The documented treatment approaches comprised dilution, anti-emetics, food or snacks, whole bowel irrigation, and intravenous fluids [8].

Symptoms of foreign body ingestion depend on the location of the obstruction ranging from dysphagia, drooling, stridor, wheezing, or a sensation of a foreign object in the throat or lower chest in cases of esophageal obstruction. On the other hand, patients with small bowel obstruction may experience nausea, vomiting, abdominal pain, distention, or tenderness [9]. A thorough history and physical examination are essential to determine the type of ingested foreign body, timing, and onset of ingestion, particularly in communicative adults. If patients exhibit signs and symptoms of aspiration, physical examinations such as pulmonary assessment for wheezing and stridor and abdominal examination for intestinal obstruction or perforation should be conducted [10]. In our case, the patient presented with nausea, vomiting, abdominal pain, and distension, with no rigidity, guarding, or rebound tenderness noted on physical examination.

All patients with suspected foreign body ingestion should initially undergo radiographic evaluation of the neck, chest, and abdomen to assess the location, size, configuration, and number of foreign objects. This helps in determining the complications such as aspiration and perforation [7,10]. However, false-negative rates are high up to 47%. While the majority of ingested foreign bodies are radiopaque, ultrasonography, CT scans, and magnetic resonance imaging (MRI) can be helpful for radiolucent objects. European Society of Gastrointestinal Endoscopy (ESGE) clinical guidelines recommend plain radiography as the initial diagnostic method for patients with suspected ingested foreign bodies without complications, and CT scans for those with suspected perforation or those requiring surgical intervention [11]. Orbeez beads, being radiolucent, can be challenging to visualize using conventional imaging techniques; therefore, a CT scan is a preferred diagnostic modality to determine the size and number of beads. In our case, the patient underwent a CT scan of the abdomen and pelvis, revealing numerous small rounded densities throughout the GIT, mostly in the stomach [9,11].

The majority of ingested foreign objects (80-90%) pass spontaneously through the GIT. Once traversed through the esophagus, most objects pass within four to six days, with rare cases taking up to four weeks [12]. However, Orbeez beads, due to their water-absorbing ability, expand in size in the digestive tract. Endoscopic retrieval is indicated in cases with water beads in the upper GIT. In cases of obstruction, surgical removal is warranted, as once the beads are stuck, it becomes challenging to remove with an increasing size over time [6,12]. Common complications associated with Orbeez bead ingestion include aspiration, GI obstruction, perforation, mucosal damage, and hemorrhage [13]. Our patient did not report signs of aspiration or perforation but complained of abdominal distension. An urgent EGD was performed, successfully removing 50-70 water beads.

## Conclusions

Orbeez beads, which are superabsorbent polymer-made beads, can pose a significant risk if ingested, as they have the potential to expand up to 200 times their original size. This property makes them a possible cause of fatal bowel obstruction, especially in children. Endoscopic retrieval is a reasonable approach if the beads are located in the esophagus or stomach, while surgical exploration is recommended in cases of perforation. We emphasize the importance of careful clinical and radiographic monitoring of patients to detect evidence of complications including obstruction or perforation.
